# Anisotropy in Bone Demineralization Revealed by Polarized Far-IR Spectroscopy

**DOI:** 10.3390/molecules20045835

**Published:** 2015-04-02

**Authors:** Roman Schuetz, Dmitri Fix, Ulrich Schade, Emad F. Aziz, Nadya Timofeeva, Richard Weinkamer, Admir Masic

**Affiliations:** 1 Department of Biomaterials, Max Planck Institute of Colloids and Interfaces, Science Park Potsdam-Golm, 14424 Potsdam, Germany; E-Mails: roman.schuetz@mpikg.mpg.de (R.S.); dmbusg@gmail.com (D.F.); nadya.timofeeva@mpikg.mpg.de (N.T.); richard.weinkamer@mpikg.mpg.de (R.W.); 2 Helmholtz-Zentrum Berlin für Materialien und Energie GmbH, Methoden der Materialentwicklung, D-12489 Berlin, Germany; E-Mails: ulrich.schade@helmholtz-berlin.de (U.S.); emad.aziz@fu-berlin.de (E.F.A.)

**Keywords:** spectroscopy, far-IR, THz, fibrolamellar bone, collagen, hydroxyapatite, dicroism, polarization, hydration, demineralization

## Abstract

Bone material is composed of an organic matrix of collagen fibers and apatite nanoparticles. Previously, vibrational spectroscopy techniques such as infrared (IR) and Raman spectroscopy have proved to be particularly useful for characterizing the two constituent organic and inorganic phases of bone. In this work, we tested the potential use of high intensity synchrotron-based far-IR radiation (50–500 cm^−1^) to gain new insights into structure and chemical composition of bovine fibrolamellar bone. The results from our study can be summarized in the following four points: (I) compared to far-IR spectra obtained from synthetic hydroxyapatite powder, those from fibrolamellar bone showed similar peak positions, but very different peak widths; (II) during stepwise demineralization of the bone samples, there was no significant change neither to far-IR peak width nor position, demonstrating that mineral dissolution occurred in a uniform manner; (III) application of external loading on fully demineralized bone had no significant effect on the obtained spectra, while dehydration of samples resulted in clear differences. (IV) using linear dichroism, we showed that the anisotropic structure of fibrolamellar bone is also reflected in anisotropic far-IR absorbance properties of both the organic and inorganic phases. Far-IR spectroscopy thus provides a novel way to functionally characterize bone structure and chemistry, and with further technological improvements, has the potential to become a useful clinical diagnostic tool to better assess quality of collagen-based tissues.

## 1. Introduction

Bone is a nanocomposite consisting of an organic collagen matrix and inorganic mineral particles of carbonated hydroxyapatite [1,2]. The amount of mineral incorporated into the bone is a crucial indicator of bone mechanical properties and bone material quality [3]. With increase in mineral content the bone gets stiffer, but less tough [4]. The reason of the varying mineral content in bone is a biomineralization process together with the renewal process of bone remodeling [5]. While the initially deposited osteoid is unmineralized, mineralization results in a constant increase of the mineral content of bone. It was demonstrated *in vitro* that a collagen fibril together with a supersaturated solution of calcium phosphate and an inhibitor (to avoid nucleation already outside the collagen fibril) is sufficient for the formation of a mineralized collagen fibril with nanosized crystal particles [6]. *In vivo* studies have clarified some of the details of early mineralization, like the transport of the ions in vesicles [7] and the formation of an initially amorphous and octacalcium phosphate precursor phase [8,9]. The opposite process of demineralization attracts interest since most of our medications against age-related bone loss typically target the bone resorption process [10,11]. Resorption by osteoclasts occurs as a multi-step process, where, first, osteoclasts seals off a region at the bone surface, in which they create a strongly acidic environment to resolve the mineral phase [12,13]. Only after demineralization is completed osteoclasts break up the collagen matrix. For *in vitro* demineralization experiments of bone typically Ethylenediaminetetraacetic acid (EDTA) is used to resolve the mineral phase while leaving the structure of the organic phase virtually untouched [14]. The timing of the exposure of bone to an EDTA solution allows producing bone with reduced mineral content and, consequently, changed mechanical properties [15,16,17].

The bone nanostructure has been intensively studied by scanning electron microscopy (SEM) and atomic force microscopy [18]. Recently the limitation of the two-dimensionality of SEM was overcome using a dual beam electron microscope [19], where a focused ion beam is used for removing a thin layer of the sample and an electron beam for imaging the new surface. After complete demineralization, the three dimensional collagen organization of lamellar bone from rat tibiae and human femora revealed distinct sub-lamellar structural motifs with strong differences in order [20,21]. A well-established method to characterize the mineral phase in bone is X-ray scattering. Small angle X-ray scattering (SAXS) provides information about the thickness, length, orientation and mutual arrangement of the mineral particles [22]. Wide angle X-ray scattering (WAXS) provides information about the crystal structure and lattice orientation of the mineral particles [23,24]. For our work relevant results are that during mineralization the mineral particles grow first in length and then in thickness [25]. A combination of synchrotron X-ray diffraction and scattering with *in situ* tensile testing demonstrated that tissue, fibrils, and mineral particles take up successively lower levels of the applied strain [26].

Fourier transform infrared (FTIR) and Raman spectroscopy have been successfully applied to obtain information about the molecular structure of bone. Being sensitive to both mineral (inorganic) and collagen (organic) matrix components of bone, it allows the studies of mineral-matrix interactions as well as each individual component’s properties [27,28,29,30,31,32]. Several material characteristics extracted from the vibrational spectra (namely mineral to matrix ratio, mineral crystallinity and maturity, lipid and proteoglycan content, collagen cross-links *etc.*) provide fundamental insights into the structure and pathophysiology of the bone [30,33]. Due to the specific arrangement of mineral platelets and collagen fibrils with respect to the main axis in case of a long bone, signals relative to vibrational units of both mineral and collagen can result highly anisotropic [34,35,36,37,38]. The polarized vibrational spectroscopy approach has been used to analyze complex three-dimensional arrangement of collagen in bone and other collagen-based tissues [39,40,41,42,43,44,45]. The aim of this work is to explore length scales that are in-between the ones typically addressed by SEM and SAXS (meso- and nano-scale), and traditional vibrational spectroscopy (chemical bonds), by using far-IR spectroscopy.

Far-IR spectroscopy, that covers the range of the electromagnetic spectrum from 50 to 500 cm^−1^, slightly overlaps with the more popular Terahertz (THz) spectroscopy that is generally restricted to 0.3–3 THz (10–100 cm^−1^) [46]. The far-IR spectrum provides structural information associated with the low frequency vibrational modes characteristic for collective motions that extend over a large portion of the molecular framework. These include collective vibrations and conformational changes of large biomolecules as well as crystal lattice vibrations. Given the long-range and collective nature of these vibrational modes they are highly sensitive to intermolecular interactions, particularly to the nature of intermolecular hydrogen bonds and thus provide a unique fingerprint of the molecular structure. In 1974, Gordon *et al.* [47] measured collagen and gelatin absorbance in the far-IR region and showed the temperature dependence of their characteristic peaks, which can be related to the higher level intermolecular assembly of the triple helix backbone of collagen. More recently, THz study of collagen based-materials (rat tail tendon, tilapia and bovine skin) confirmed the earlier identification of the absorption peaks in the far-IR spectral range between 100–600 cm^−1^ [48,49]. Far-IR spectroscopy in various spectral ranges was also applied to study bone material, e.g. for monitoring senile osteoporosis development [50], even though most of the studies on bone have been performed within the spectral window below 100 cm^−1^ [51]. Characterization of different minerals by far-IR spectroscopy [52,53] showed remarkable spectral differences for different carbonate minerals, which can serve as fingerprints for mineral identification. 

We employed far-IR spectroscopy to address questions on the structure of the mineral/water/protein matrix of the bone material. Our far-IR measurements have been performed in transmission geometry. Due to the small sample size and the complex sample chamber a small focus of high intensity is required to gain sufficient signal-to-noise in a reasonable period of time with constant environmental conditions for the fully mineralized and demineralized bone samples. To overcome the limitations of conventional thermal infrared sources synchrotron radiation from an electron storage ring [54] was used to illuminate the sample with a diffraction limited far-IR spot in the order of some hundred micrometer.

The aim of this study is to fathom the potential of far-IR spectroscopy to study the structural interplay between the collagen matrix and the mineral particles in bone. Our current understanding is that in fully mineralized bone the collagen matrix is strongly confined by the surrounding “mineral cage” Performing stepwise demineralization experiments, we ask the question whether changes in the spectrum of the mineral phase with proceeding demineralization and whether we can observe a “freeing” of collagen due to the reduction in mineral content. Far-IR spectroscopy seems a promising method to address these structural changes. For our studies we use bovine fibrolamellar bone as model system since this bone is known to have a well ordered structure with a preferred orientation of both the collagen matrix and the mineral particles along the axis of the long bone [55,56]. This structural anisotropy is addressed by using a polarized beam with orthogonal planes of polarization evaluating the vibrational properties parallel and perpendicular to the predominant orientation of the collagen fibers. 

## 2. Results

### 2.1. Design of a Sample Chamber for Hydrated Biological Samples

**Figure 1:**
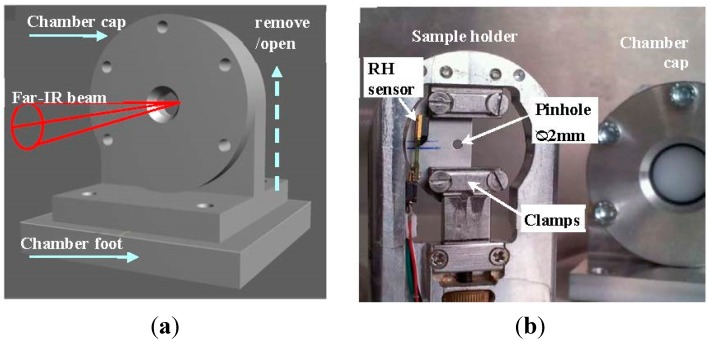
Sample chamber for far-IR measurements of biological samples in controlled environmental conditions. (**a**) Schematic drawing of the sample chamber with the chamber cap fixed on the chamber foot. The humid air is introduced into the chamber from outside though drilled pipes in the chamber foot; (**b**) The sample holder (on the left) and the removable chamber cap (in the back right side) are shown. The sample fixed with clamps can be stretched using a micrometer screw. Behind the sample a pinhole is positioned to narrow the beam spot and, thereby, significantly improving the spectral quality. A digital humidity and temperature sensor (RH sensor) is positioned close to the sample.

Biological materials are generally evolutionary adapted to function in hydrated and or humid environments. However, when it comes to measuring in the far-IR region, strong absorption bands of water in that region present a significant technical challenge. Normally, these issues are solved by performing the experiments in vacuum or very dry conditions. In the case of bone samples, however, this is not a viable option considering the amount of stress generated by drying collagen molecules [57]. To obtain spectroscopic information from bone samples in conditions that are as close as possible to the physiological ones, a sample chamber was designed and built with the aim to keep the sample in a small, humidity controlled environment while the rest of the optical path is in vacuum. The designed sample chamber (Figure 1) is equipped with interchangeable diamond and high-density polyethylene windows with an area of each window of 4.52 cm^2^ and a window-to-window distance of 5 mm. The construction of the small chamber with the windows very close to the sample ensures a minimal beam path through the humid environment. The chamber is equipped with a mini-sensor measuring the humidity close to the sample. All experiments were performed in a controlled humidity environment.

### 2.2. Far IR Spectra of Bone and Its Components (Hydroxyapaptite and Collagen)

**Figure 2:**
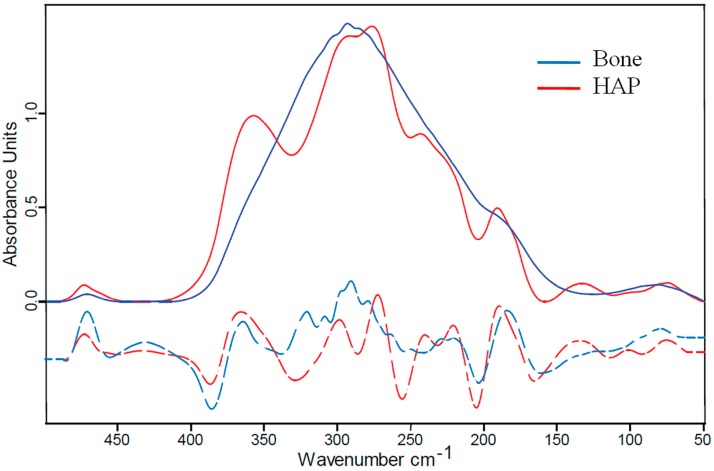
Far-IR spectroscopy on bone. The spectrum of a 18 µm-thick foil of fibrolamellar bovine bone (**blue line**) is shown together with the spectrum of hydroxyapatite (HAP) powder (**red line**), HAP being the mineral component of the nanocomposite bone. Both samples absorb the radiation in a very similar spectral region and, apart from peak broadening in the case of bone, show similar spectral features evidenced also through the second derivative analysis (**dashed lines**).

Figure 2 shows the far-IR spectrum obtained with a 18 µm thick bone sample (blue line). The spectrum is characterized by a strong absorption in the range from 150–400 cm^−1^, which can be assigned to apatite lattice modes, and a smaller peak at about 470 cm^−1^ assignable to the υ_2_ PO_4_^3−^ vibration [58]. The strong absorption band has its maximum at 293 cm^−1^ and is further characterized by shoulders at 190, 230, 320 and 360 cm^−1^. For comparison, the far-IR spectrum of the hydroxyapatite (HAP) powder (red line) is superimposed in the Figure 2. Also HAP absorbs in the spectral region from 150–400 cm^−1^ but its spectral features are more prominent with respect to the bone spectrum and, instead of shoulders, it has separated peaks at 190, 242, 276, 293, and 360 cm^−1^.

### 2.3. Far-IR Spectroscopy of Bone Demineralization

**Figure 3:**
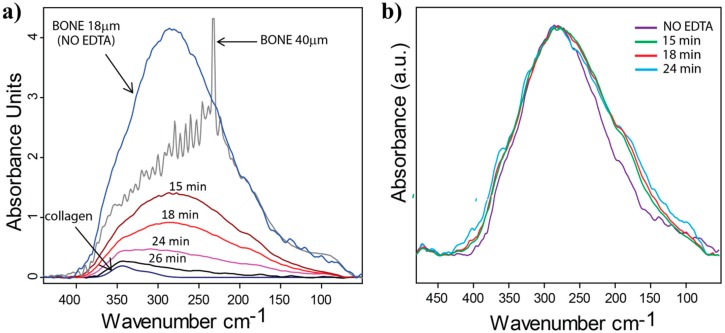
Far-IR spectroscopy at different stages of demineralization of fibrolamellar bovine bone. (**a**) the 18 µm-bone spectrum (fully mineralized, blue line), is plotted together with the 40 µm thick sample (grey line) and the series of spectra related to the demineralization steps (15, 18, 24, 26 and 30 min of EDTA treatment). The time (min) denotes the total time the sample was immersed into the EDTA solution. The sample was removed from the EDTA solution and washed in distilled water before the measurement, which was performed under humid conditions (relative humidity 95%); (**b**) calculated mineral spectra (after the collagen contribution is subtracted and normalized to their maxima) for different steps of demineralization (after 15, 18 and 24 min and fully mineralized 18 µm thick sample). Very similar spectral features originating from the mineral phase of bone can be observed throughout the entire demineralization process.

For the demineralization experiment, in order to ensure the integrity of the sample during the treatment with EDTA, a thicker bone sample (40 µm; grey line) was used. The stronger absorption of the thicker sample leads, though, to a saturation in the range of 200–350 cm^−1^ in the absorbance spectrum. After immersing the sample for 15 min in a 1M solution of EDTA, the mineral content is reduced so that the spectrum does not suffer from saturation and displays very similar spectral features compared to the 18 µm thick sample (superimposed in Figure 3a and normalized to the part of the spectrum of the 40 µm thick sample that is not saturated). The spectral features do not significantly change even after 18 and 24 min of EDTA treatment, only the absorbance is further reduced. After 30 min of EDTA treatment all mineral is removed and the spectrum (dark blue line) is characterized by two collagen peaks centered at 315 and 345 cm^−1^ [47].

In order to analyze more accurately the spectral features of the mineral that is dissolved during the demineralization process, first the collagen contribution was subtracted from all the measured spectra (Figure 3b), and second, the “mineral loss” can be obtained by subtracting the spectra of two successive demineralization steps. For the evaluation we assumed that the concentration of each component of bone (mineral, collagen, water) contributes linearly to the absorbance. We further assumed that the collagen contribution into the absorbance spectra remained constant for the different demineralization steps. In order to estimate the pure mineral contribution, the collagen spectrum, which was measured after full demineralization of the same bone sample, was subtracted from the total absorbance profile peak. Calculated spectra free of the contribution of collagen for different steps of demineralization are compared to the mineral only spectrum of fully mineralized bone sample (thickness 18 µm, see also Figure 2). The minor differences observed in the spectra throughout the entire demineralization process were not found to be significant within the limits of our analytical scheme. This suggests a gradual and homogeneous removal of the bone mineral.

### 2.4. Far-IR Linear Dichroism of Bone and Bone Mineral 

**Figure 4:**
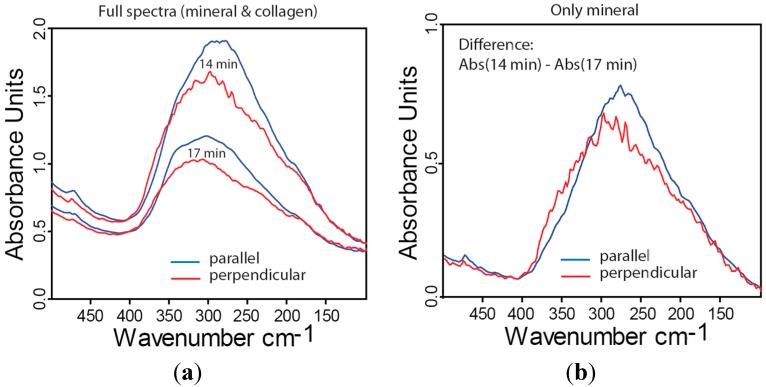
Far-IR dichroism measurements of bovine fibrolamellar bone showing the absorbance parallel (**blue**) or perpendicular (**red**) to the long axis of the bone. (**a**) Spectra obtained after two different time intervals of demineralization (14 and 17 min total time in EDTA); (**b**) Calculated spectra after subtracting the contribution of collagen (see text in Section 2.3 for an explanation of the evaluation) therefore only reflecting the contribution of the mineral phase to the absorbance. The structural anisotropy is clearly reflected in anisotropic absorption properties.

Fibrolamellar bone is composed of mineralized collagen fibers with a predominant orientation along the long axis of the bone [55]. Consequently, this structural anisotropy should be reflected in an anisotropic absorbance when polarized radiation is used. In Figure 4a the polarized far-IR spectra of partly demineralized lamellar bone are shown after two steps of demineralization (14 and 17 min in EDTA). Blue lines denote the spectra acquired with an incident beam polarized parallel to long axis of the bone (*i.e.*, parallel to the predominant collagen orientation) and red lines are spectra obtained with a polarization perpendicular to this orientation. The parallel configuration shows higher absorbance indicating the preferential orientation of vibrational units along the collagen fiber. Performing a similar evaluation strategy in subtracting the collagen contribution as described in the context of Figure 3b, but now subtracting two spectra taken at consecutive demineralization step under the same polarization conditions (Figure 4b). The comparison demonstrates that also the vibrations of the mineral phase are stronger when the IR beam is parallel to bone main axis.

### 2.5. Far-IR Linear Dichroism of Collagen from Fully Demineralized Bone

**Figure 5:**
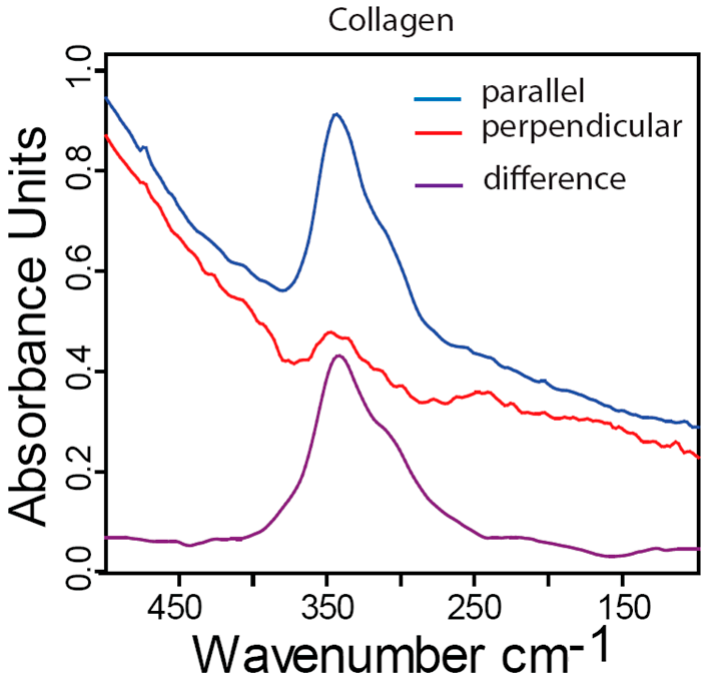
Far-IR dichroism measurements of fully demineralized fibrolamellar bovine bone showing the absorbance parallel (**blue**) or perpendicular (**red**) to the long axis of the bone. The violet spectrum denotes the difference between these two spectra.

To isolate the contribution of the collagen matrix phase to the absorption spectrum, the bone samples required more than 30 min 1M EDTA treatment in order to dissolve the entire mineral component. Although the spectrum of collagen in far-IR region was reported already four decades ago [47], the response of the tissue to polarized light is still unknown. Figure 5 shows far-IR linear dichroism measurements of fully demineralized fibrolamellar bone. Characteristic collagen peaks show very high anisotropy and the absorbance vanish almost completely when the polarization of the incident IR beam is 90 degrees with respect to the collagen fiber orientation. Since the beam size (approximately 1.7 mm) is much larger than the typical size of a of a fibrolamellar bone unit (about 200 µm), our measurements also include less organized interface regions that are separating the bone units. Although these regions create some “unordered background” in the spectra, the contribution of all the fibrolamellar bone units provides a clear preferred direction of collagen vibrational units.

### 2.6. Influence of Hydration and Stress on Far-IR Spectra of Collagen

**Figure 6:**
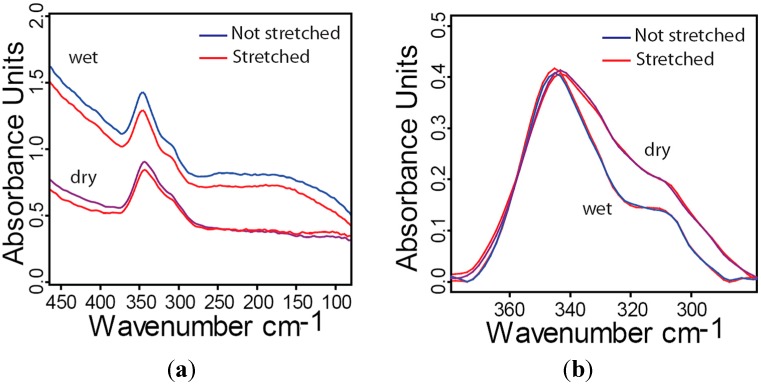
Far-IR spectra of collagen from fully demineralized bovine fibrolamellar bone. (**a**) Comparison between the spectra of stretched (8%–10% strain, red) to unstretched and under wet and dry conditions; (**b**) Same spectra but baseline corrected and “zoomed in” to focus on wavenumbers between 380 and 280 cm^−1^. Absorbance peaks are normalized to their maximum.

To study the influence of mechanical stresses and/or dehydration on the absorbance of collagen in the far-IR region a fully demineralized fibrolamellar bone sample was loaded under tension in the sample chamber under controlled humidity (see description of the controlled environment chamber in the experimental Section 4). The sample was orientated in such a way that the loading direction was aligned with the preferential orientation of the collagen fibers. The same sample was first measured under wet (relative humidity approximately 95%) and then in dry conditions (relative humidity lower than 10%) without additional load. After humidifying the sample, it was stretched for about 8%–10% of its initial length and measured again, first in wet and afterwards in dry conditions. All four obtained spectra are shown in Figure 6.

No differences in spectral features due to the externally applied stress can be observed. The hydration level, on the other hand, has effects on the far-IR absorbance features for wavenumbers below 340 cm^−1^. Drying the sample affects the intensity ratio between the two collagen peaks at 315 and 345 cm^−1^ suggesting conformational changes of the collagen backbone.

## 3. Discussion

We measured for the first time far-IR spectra of bone where stepwise demineralization with EDTA allowed studying changes in the spectrum as a function of the mineral content. Exposure to EDTA is the standard procedure to demineralize bone. It is commonly assumed that the mineral matrix dissolves in a similar way than in the living bone during bone remodeling. Our experiments allowed four particularly interesting observations that are discussed in the following: 

(1) From the 2nd derivative analysis it results that peaks of the synthetic hydroxyapatite powder (HAP) and the fibrolamellar bone are at similar positions. The spectral differences between HAP and the bone mineral phases can be related to the chemical differences, since the mineral phase in bone is known to deviate from the crystalline hydroxyapatite (by carbonization) [20,45,58,59]. However, more relevant to the broadening seems the difference in mineral size. Where in the powder the crystallites have an average size in order of micrometers, the mineral particles in bone have a thickness of only a few nanometers [2]. These small dimensions imply also structural changes as a significant part of the crystal units are at the surface with reduced crystalline order compared to the bulk. Our interpretation of the smearing out of the spectrum of the mineral phase in bone is due to this nanosize of the particles. 

(2) The spectrum of completely unmineralized bone is very similar to spectra of type I collagen (results not shown) demonstrating that demineralization removes the mineral phase without visible alterations in the spectrum of collagen. The dichroism experiments of fully demineralized bone underline this point, since they show the clear anisotropic response of the triple-helical structure of collagen I (Figure 5). 

(3) We did not observe changes in the spectrum while applying a tensile strain of roughly 9% on the sample. An explanation could be that in wet samples only a minor part of the externally applied strain arrives at the small length scale of the collagen molecule since sliding between structural units leads to strain reduction [26,60,61]. 

(4) The latter observation can partly explain why the measured spectra of bone at different steps of demineralization can be intended as a simple addition of the collagen spectrum and the spectrum coming from the mineral phase (Figure 3). In any case, it should be kept in mind that demineralization probably does not occur homogeneously throughout the sample. While mineral particles get already completely dissolved close to the surface, others in the bulk of the sample most likely only start to dissolve. As a consequence, the measurement always characterizes a mixture of particles in different states of dissolving. 

(5) Polarized IR study of demineralization steps showed intensity changes in spectra collected along and perpendicular to the axis of the long bone. Already a single spectrum is different in both directions (Figure 4a) which can be associated with the different dimensions of the length and thickness of the mineral particles. Furthermore, the polarized spectral features of different demineralization steps result different (Figure 4b) suggesting that demineralization changes the aspect ratio of the mineral particles. For mineralization process, a similar scenario with an initial growth in length followed by an increase in thickness was detected with SAXS [25].

When it comes to the limitations of the proposed characterization method the collection and the interpretation of far-IR spectra is very challenging. For the interpretation computer simulations, most adequately Molecular Dynamic simulations, could help in the better interpretation [58]. In addition, the diagnostic potential of the proposed method has to be tested in the future. Up to now, it is not clear, if differences in healthy and diseased bone—at least for bone diseases with known alterations in the organization of the organic matrix or the mineral particles—can be detected. A further limitation of our method towards diagnostic applications is that our results are restricted to transmission measurements. If measurements performed in reflectance geometry collect the same information on the bone structure, handheld diagnostic instrumentations could be developed in the future employing modern spectroscopic techniques with far-infrared quantum cascade lasers [62,63]. Further improvements in the measurement technology, but especially in the interpretation of far-IR spectra have to prove the potential of this method to offer new insights into the hierarchical structure of collagen-based materials and to open new possibilities for the diagnostic of bone diseases. 

## 4. Experimental Section

### 4.1. Far-IR at BESSY

The experiments were conducted at the IRIS beamline of Berlins Synchrotron Radiation Facility (BESSYII) at Helmholtz-Zentrum Berlin (HZB) [54]. Strong and brilliant IR-Synchrotron Edge Radiation (ED) was used as a source for the measurements [64]. For the experiments in the far-IR range of (30–600 cm^−1^) FT-IR IFS 66v Bruker Spectrometer was used with 50 and 125 µm Mylar beam splitter. On the detection side the spectrometer was equipped with a liquid He cooled Si-Bolometer 4.2K (spectral range 10–600 cm^−1^).

The dichroism measurements were performed with an additional polarizer, which was placed in the beam path before the last mirror that focuses the far-IR beam onto the sample inside the sample chamber. For polarization a wire grid polarizer on a supporting foil consisting of Mylar, which is actually polyethylenterephthalat (PET), was used.

### 4.2. Controlled Environment Sample Chamber

We have developed a sample chamber equipped interchangeably with (1) diamond or (2) high-density polyethylene windows. (1) First chamber-cup windows: CVD Diamond with a good transparency of a big spectral range of 25.000–33 cm^−1^ but with a relatively small diameter. (CVD Diamond Window, Diafilm OP; dimensions: diameter: 15.0 ± 0.1 mm; thickness: 0.5 ± 0.05 mm; flatness: <5 fringes@632.8 nm in reflection; roughness: <15 nm Ra measured on 1 mm^2^; wedge: 1°); (2) The second cup was equipped with bigger windows of 24 mm diameter made out of high density polyethylene, which has a good transparency in the far-IR region of 600–30 cm^−1^. In this study only measurements conducted with the bigger high density polyethylene windows are presented. 

The construction of the small chamber with the windows close to the sample ensures a minimal path of the beam through a non-evacuated environment. The chamber is equipped with a mini-sensor measuring the humidity. A SENSORION SHT75 digital humidity and temperature sensor was fixed on the inner frame edge of the sample holder only a few millimeters away from the sample (Figure 1). The sensor values were readout by the evaluation kit EK-H4 (fa. Sensorion).

All experiments were performed in controlled environmental conditions. The atmosphere inside the chamber can be controlled from outside by conducting the gas medium via two tubes connected to the holes drilled into the chamberfoot. 

### 4.3. Sample Preparation and Measurement Procedure

Thin (20 to 100μm) samples were extracted from the subperiosteal fibrolamellar region of the mid-diaphysis of the femur from a 12 month old calf. The bone was cut in the radial-longitudinal sections parallel to the long axis with an inner-hole saw (Leica SP1600, Leica Mikrosystem Vertrieb GmbH, Bensheim, Germany). The slides were then polished by means of an automatic polisher (Logitech PM5, Logitech Ltd., Glasgow, UK) with 3 μm and 1 μm grit-sized diamond particles (DP-Spray P, Struers A/S, Ballerup, Denmark) until the samples achieved the right thickness. Before the measurements the bone foils were cut in 6 mm wide and 5–10 mm long stripes along the fibrolamellar orientation. For storage the samples were kept at −20 °C wrapped by 1% sodium azide in phosphate buffered saline (PBS) solution soaked gauze. 

Bone samples, thicker than 20 μm, required demineralization in order to give an unsaturated far-IR spectrum because of the strong absorption by mineral particles. The demineralization was done by means of Ethylenediaminetetraacetic acid—EDTA (0.5 M and 8 Ph) solution. Two ends of the thin bone sample were first glued on a plastic frame before immersing them into the EDTA solution. The subsequent fixing in the environmental chamber or optionally further demineralization was done with the plastic frame as well in order not to rupture the thin demineralized samples.

For the stepwise demineralization series the sample was immersed into EDTA solution for 3–5 min before measurement. After the measurement, the sample was immersed again and subsequently washed in distilled water before further measurements. For stretching experiments the fully demineralized samples were fixed between clamps while still glued onto the plastic frame. After clamping the plastic frame were cut through to allow the stretching of the sample. The distance between the clamps can be increased manually by means of the micrometer screw on the lower clamp side (Figure 1).

Measurements performed on laterally narrow samples often showed a strong spectral modulation pattern corrupting the measured spectrum. We assume that the reason for this unwanted signal were small holes present in the thin samples or the beam hitting a crack or an edge of the sample. To avoid this it was necessary to cut down the spot size of the beam on the sample. Therefore, measurements were conducted with an additional pinhole of 2 mm in diameter on a metal plate, which was fixed behind the sample on the upper clamp. The lower clamp was still moveable by the micrometer screw. The pinhole on one hand narrows and sharpens the beam profile significantly, but on the other hand it causes additional spectral modulation. This additional modulation could be subtracted from the final spectra after the absorption of pinholes have been characterized without the sample. The background absorbance was determined in a common way at the empty side of the pinhole plate passing through the windows and the atmosphere of the environmental chamber between them. 

### 4.4. Data Evaluation

In this work multi composite materials like fully mineralized or partly demineralized bone were measured and a linear compound model for spectral absorption analysis was used. In our bone samples collagen type I, hydroxyapatite plates and water are the main composites, whose variety in density, distribution and proportion can influence the properties of absorption. 
